# ROS-mediated inactivation of the PI3K/AKT pathway is involved in the antigastric cancer effects of thioredoxin reductase-1 inhibitor chaetocin

**DOI:** 10.1038/s41419-019-2035-x

**Published:** 2019-10-24

**Authors:** Chuangyu Wen, Huihui Wang, Xiaobin Wu, Lu He, Qian Zhou, Fang Wang, Siyu Chen, Lanlan Huang, Junxiong Chen, Huashe Wang, Weibiao Ye, Wende Li, Xiangling Yang, Huanliang Liu, Junsheng Peng

**Affiliations:** 1 Guangdong Provincial Key Laboratory of Colorectal and Pelvic Floor Diseases, Guangdong Institute of Gastroenterology, The Sixth Affiliated Hospital, Sun Yat-sen University, Guangzhou, Guangdong China; 2 Department of Gastrointestinal Surgery, The Sixth Affiliated Hospital, Sun Yat-sen University, Guangzhou, Guangdong China; 3 Department of Clinical Laboratory, The Sixth Affiliated Hospital, Sun Yat-sen University, Guangzhou, Guangdong China; 4 Guangdong Laboratory Animals Monitoring Institute, Guangdong Key Laboratory Animal Lab, Guangzhou, Guangdong China; 5 Dongguan Hospital of Southern Medical University, Guangzhou, Guangdong China; 6 School of Nursing, Sun Yat-sen University, Guangzhou, Guangdong China

**Keywords:** Gastric cancer, Natural products, Pharmacodynamics

## Abstract

Novel drugs are urgently needed for gastric cancer (GC) treatment. The thioredoxin-thioredoxin reductase (TRX-TRXR) system has been found to play a critical role in GC tumorigenesis and progression. Thus, agents that target the TRX-TRXR system may be highly efficacious as GC treatments. In this study, we showed that chaetocin, a natural product isolated from the *Chaetomium* species of fungi, inhibited proliferation, induced G_2_/M phase arrest and caspase-dependent apoptosis in both in vitro and in vivo models (cell xenografts and patient-derived xenografts) of GC. Chaetocin inactivated TRXR-1, resulting in the accumulation of reactive oxygen species (ROS) in GC cells; overexpression of TRX-1 as well as cotreatment of GC cells with the ROS scavenger N-acetyl-L-cysteine attenuated chaetocin-induced apoptosis; chaetocin-induced apoptosis was significantly increased when GC cells were cotreated with auranofin. Moreover, chaetocin was shown to inactivate the PI3K/AKT pathway by inducing ROS generation; AKT-1 overexpression also attenuated chaetocin-induced apoptosis. Taken together, these results reveal that chaetocin induces the excessive accumulation of ROS via inhibition of TRXR-1. This is followed by PI3K/AKT pathway inactivation, which ultimately inhibits proliferation and induces caspase-dependent apoptosis in GC cells. Chaetocin therefore may be a potential agent for GC treatment.

## Introduction

Gastric cancer (GC) is the fifth most common cancer and the third leading cause of cancer-related death worldwide^1^. The traditional treatment options for GC include surgery and chemotherapy. However, the 5-year survival rates of patients receiving these therapies are still very low. The targeting of specific molecules could be an efficient strategy for the treatment of GC. Recently, some novel molecular targeted agents have been used to treat GC, including trastuzumab (which targets the epidermal growth factor receptor) and bevacizumab (which targets the vascular endothelial growth factor). However, the efficiency of these drugs is limited^2,3^. Thus, the identification of new molecular targets and the development of novel targeted therapeutic agents, which can be highly efficacious in treating GC, are urgently needed.

The thioredoxin-thioredoxin reductase (TRX-TRXR) system, which is composed of TRXRs, TRXs and NADPH, plays an essential role in maintaining cellular redox homeostasis. In eukaryotes, the flavoenzyme TRXRs have two confirmed forms, TRXR-1 and TRXR-2, which are located in the cytoplasm and mitochondria, respectively. TRXRs are the only known TRX-reducing enzymes; they generate reduced TRXs by providing reducing equivalents in an NADPH-dependent reaction. Reduced TRXs function as antioxidants by donating reducing equivalents to reactive oxygen species (ROS) scavenging enzymes such as peroxiredoxins. By regulating cell redox events, the TRX-TRXR system influences various cellular functions including proliferation, differentiation and death^4–7^. However, the TRX-TRXR system has recently been found to be upregulated in a variety of human cancers including gastric, colorectal, lung and liver cancers, and overexpression of specific components of this system is linked to tumor cell proliferation, invasion, metastasis, and drug resistance^8–11^. Inhibition of the TRX-TRXR system abolishes tumor progression^12–14^. As the TRX-TRXR system plays an important role in GC tumorigenesis and progression^11,13^, targeting the TRX-TRXR system may represent an effective GC treatment option.

Chaetocin is a small-molecule thiodioxopiperazine natural product isolated from the *Chaetomium* species of fungi^15,16^. Recently, some studies have shown that chaetocin has a potent inhibitory effect on cancer cells^17–21^, indicating that chaetocin may be a potential agent for cancer therapy. Molecular mechanisms associated with the anticancer effect of chaetocin are still vague. The inhibition of histone methyltransferase suppressor of variegation 3–9 homolog 1 (SUV39H1), which trimethylates lysine 9 of histone h3, and hypoxia-inducible factor-1α (HIF-1α) may be included in the anticancer activity of chaetocin^22–24^. Most importantly, chaetocin was shown to inhibit the activity of TRXR-1 in the cell-free system, which may be related to its anticancer effect^25^. However, the pharmacological effect and underlying mechanism of action of chaetocin in GC cells remains unclear. In the present study, we investigated the antiGC effects of chaetocin both in vitro and in vivo and determined whether chaetocin exerts its anticancer effects in GC by inhibiting TRXR-1.

## Materials and methods

### Cell culture

Human gastric cancer cell lines HGC-27, AGS, BGC-823, SGC-7901 and human embryo kidney cell line HEK-293T were purchased from the Culture Collection of the Chinese Academy of Science (Shanghai, China). Human gastric cancer cell lines SNU-216, MKN-45 and human gastric mucosa epithelial cell line GES-1 were obtained as a gift from Professor Ruihua Xu, State Key Laboratory of Oncology in South China, Sun Yat-sen University Cancer Center. HEK-293T cells were maintained in DMEM (Life Technologies, Carlsbad, CA, USA), and all other cell lines were maintained in RPMI 1640 (Life Technologies). All culture media were supplemented with 10% fetal bovine serum (Life Technologies), 100 units/ml penicillin and 10 mg/ml streptomycin (Life Technologies). All cells were cultured in a humidified 5% CO_2_ atmosphere at 37 °C.

### Reagents

Chaetocin was purchased from Sigma-Aldrich (St. Louis, MO, USA). Chaetocin was resuspended in DMSO at a concentration of 10 mM and was stored at −20 °C. z-VAD-fmk (Selleck Chemicals, Houston, TX, USA) was resuspended in DMSO at a concentration of 100 mM and was stored at −20 °C. LY294002 (Selleck Chemicals) was resuspended in DMSO at a concentration of 50 mM and was stored at −20 °C. N-acetyl-L-cysteine (NAC) (Sigma-Aldrich) was resuspended in DMSO at a concentration of 0.5 M and was stored at −20 °C. phospho-histone h3 (Ser473), phospho-CDK1 (Thr161), PARP, caspase-3, cleaved-caspase-3, caspase-9, cleaved-caspase-9, caspase-8, BCL-2, BCL-XL, MCL-1, survivin, XIAP, TRX-1, phospho-AKT (Ser473), AKT and ki-67 antibodies were purchased from Cell Signaling Technology (Beverly, MA, USA). β-actin and flag tag antibodies were purchased from Proteintech Group (Chicago, IL, USA). Anti-mouse immunoglobulin G and anti-rabbit immunoglobulin G horseradish peroxidase-conjugated secondary antibodies were purchased from Sigma-Aldrich.

### TRX-1 and AKT-1 overexpression

A pLV-EF1α-EGFP(2A)Puro vector with TRX-1 insert was purchased from Cyagen Biosciences (Suzhou, Jiangsu, China) and used to stably overexpress TRX-1. Expression, packaging (psPAX2) and envelope (pMD2.G) plasmids were transfected into HEK-293T cells using lipofectamine 3000 (Life Technologies). Lentiviral particles were collected from the supernatant and used to infect HGC-27 and AGS cells. Stable cell lines were established by puromycin selection. A pENTER-Flag vector with AKT-1 insert was purchased from Vigene Biosciences (Jinan, Shandong, China) and used to transiently overexpress AKT-1. The plasmid was transfected into HGC-27 and AGS cells using lipofectamine 3000 (Life Technologies). A total of 24 h after transfection, AKT-1 expression levels in HGC-27 and AGS cells were confirmed by western blot, and transfected cells were used for subsequent experiments.

### Real-time cell impedance analysis

The xCELLigence system (Roche Applied Science, Mannheim, Germany) was used to dynamically monitor cell proliferation rates. Experiments were performed using a standard protocol developed by Roche Applied Science. Briefly, HGC-27 and AGS cells were seeded into 100 µl of media in an E-Plate. Cell proliferation was monitored by measuring electrical impedance across microelectrodes on the bottom of the E-Plate. Impedance was expressed as the normalized cell index, which is an arbitrary unit. The results were analyzed using the real-time cell analysis software supplied by the company.

### Cell viability assay

A cell counting kit-8 (CCK-8) assay (Nanjing KeyGen Biotech Co., Ltd.) was used to analyze the effect of chaetocin on GC cell viability. Briefly, 100 µl of 1 × 10^5^/ml cells were treated with various doses of chaetocin for 24 h. Then, 20 µl CCK-8 reagents were added to each well and incubated for an additional 4 h. The absorbance was measured at 450 nm using a Varioskan Flash multimode reader (Thermo Fisher Scientific, Waltham, MA, USA).

### Colony formation assay

HGC-27 and AGS cells were seeded in a 6-well plate (500 cells/well) and treated with various concentration of chaetocin. Cells were cultured in a 5% CO_2_ atmosphere at 37 °C for 9 d, and the culture medium was changed every 3 d. After chaetocin treatment, cells were washed with PBS, fixed in ice-cold methanol, and stained with crystal violet. An Epson scanner (Suwa, Nagano, Japan) was used to image the colonies.

### Cell cycle analysis by flow cytometry

After chaetocin treatment, HGC-27 and AGS cells were collected, washed and fixed with cold 66% ethanol overnight at 4 °C. Cells were then washed in PBS and labeled with 500 µl propidium iodide (PI) (BD Biosciences, Franklin Lakes, NJ, USA). The FACSCanto II flow cytometry (BD Bioscience) was used to analyze cell cycle distribution.

### Cell apoptosis analysis by flow cytometry

An annexin V-FITC/PI staining kit (Nanjing KeyGen Biotech Co., Ltd.) or an annexin V-APC/7-aminoactinomycin D (7-AAD) staining kit (MultiSciences (Lianke) Biotech Co., Ltd., Hangzhou, Zhejiang, China) was used to evaluate apoptosis. Following chaetocin treatment, cells were collected, washed and stained in working solution (500 µl binding buffer with 5 µl annexin V-FITC and 5 µl PI or 500 µl binding buffer with 5 µl annexin V- APC and 10 µl 7-AAD) for 15 min at room temperature in the dark. The FACSCanto II flow cytometry (BD Bioscience) was used to detect apoptotic cells. Annexin V-FITC^+^ and annexin V-FITC^+^-PI^+^ cells or annexin V-APC^+^ and annexin V-APC^+^7-AAD^+^ cells were considered apoptotic cells.

### Western blot analysis

Following chaetocin treatment, HGC-27 and AGS cells were lysed in RIPA buffer (Cell Signaling Technology) containing protease and phosphatase inhibitors (Nanjing KeyGen Biotech Co., Ltd.). The concentration of each protein sample was measured using the Pierce BCA protein assay kit (Thermo Fisher Scientific, Waltham, MA, USA). Total cellular proteins were separated by SDS-PAGE and then transferred to PVDF membranes. After blocking in 5% non-fat dry milk, membranes were probed with primary antibodies overnight at 4 °C. The next day, membranes were washed with PBST, incubated with a horseradish peroxidase-conjugated secondary antibody and finally detected using enhanced chemiluminescence.

### TRXR activity assay

TRXR activity was detected using the TRXR assay kit (Abcam, Cambridge, MA, USA) according to the manufacturer’s protocol. Briefly, after chaetocin treatment, HGC-27 and AGS cells were lysed in assay buffer (provided with the kit), and the concentration of each protein sample was determined using the Pierce BCA protein assay kit (Thermo Fisher Scientific). A total of 100 μg of each protein sample was mixed with DTNB and NADPH, and the TRXR activity was calculated as the increase in absorbance of TNB^2−^ at 412 nm from 1 min to 20 min. The TRXR activity is shown as % of control.

### Determination of reduced and oxidized forms of TRX-1 in GC cells

Reduced and oxidized forms of TRX-1 in GC cells were determined as previously described^26^. After chaetocin treatment, HGC-27 and AGS cell lysates were denatured and precipitated by trichloroacetic acid at a final concentration of 7.5% and centrifuged at 12,000 g for 10 min at 4 °C to collect protein precipitates. After washed with acetone twice, the protein precipitates were solubilized in a buffer containing 50 mM Tris-HCl (pH 7.4), 1% sodium dodecyl sulfate, and 15 mM 4-acetamido-4′-maleimidylstilbene-2,2′-disulfonic acid (AMS). The reduced and oxidized forms of TRX-1 was separated by non-reducing SDS-PAGE followed by western blot analysis.

### Molecular docking simulation

The docking interaction between chaetocin and TRXR-1 was simulated using the AutoDock 4.2. The human TRXR-1 crystal structure (PDB code: 2ZZC), which has a resolution of 2.6 Å, was retrieved from the RCSB protein data bank and used for docking studies. The structure of chaetocin was assembled using Chemoffice. The binding location was defined on the active binding site of TRXR-1^27^, and the grid box (Center: −37.844, 16.961, −41.324 Å) was set to 20 × 22 × 20 Å in the X, Y, and Z directions, with spacing within a 9 Å radius.

### Molecular dynamics (MD) simulation

The complex of TRXR-1 protein and chaetocin obtained from the docking simulation were used as the initial coordinates for MD simulations. GROMACS 5.1.4 was used for MD. TRXR-1 and chaetocin were assessed using ff99sb force field and general amber force field, respectively. A truncated octahedron box of transferable intermolecular potential for water molecules with a margin distance of 10 Å, periodic boundary conditions and neutralizing counterions was added to the system. The production MD simulation was performed for 15 ns using the NPT ensemble at a temperature of 300 K, and coordinates were saved every 1 ps. The binding free energies of the chaetocin-TRXR-1 interaction were calculated using the molecular mechanics/poisson-boltzmann surface area (MM/PBSA) method.

### RNA sequencing (RNA-seq)

RNA was sequenced and analyzed by Huada (Wuhan, Hubei, China). Briefly, RNA was extracted from non-treated AGS cells as well as AGS cells with 100 nM chaetocin treatment for 12 h. RNA-seq was performed on the BGISEQ-500 platform. Reads were aligned to the human RefSeq hg38 reference genome using Bowtie2. Differentially expressed genes (DEGs) were calculated by RNA-seq by expectation maximization and possion distribution and DEGs defined as +/− twofold change and false discovery rate (FDR) < 0.01 were analyzed for enriched gene pathways using KEGG pathway analysis (http://www.genome.jp/kegg/pathway.html). *q* value of the pathway shown in the figure was <0.05.

### Determination of cellular oxygen species (ROS)

Cellular ROS production was measured using a ROS assay kit (Beyotime, Shanghai, China) according to the manufacturer’s protocol. Briefly, a total of 1.5 × 10^5^/ml cells in 2 ml was seeded in 6-well dishes. Cells were incubated with 10 µM 2′,7′-dichlorofluorescin diacetate (DCFH-DA) for 20 min in the dark and then treated with chaetocin. Cells were then washed with culture media (without fetal bovine serum) and DCFH-DA florescence was measured at 525 nm using a FACSCanto II flow cytometry (BD Bioscience).

### Immunohistochemistry

Xenograft tumors were fixed in a 4% formaldehyde solution in PBS, embedded in paraffin and sectioned. Following deparaffinization in xylene and hydration with decreasing concentrations of alcohol, sections were incubated with 0.3% hydrogen peroxide to block endogenous peroxidase activity and boiled in EDTA buffer (pH = 8.0) for antigen retrieval. Sections were then incubated with a mouse monoclonal ki-67 antibody at 4 °C overnight in a moist chamber. The next day, sections were washed with PBS, incubated with a horseradish peroxidase-conjugated secondary antibody and finally detected using 3,3′-diaminobenzidine.

### Animal model

We used five-week-old male BALB/c nude mice bred at Experimental Animal Center of Guangdong Laboratory Animals Monitoring Institute for HGC-27 xenograft model. A total of 1 × 10^7^ HGC-27 cells were subcutaneously injected into the flanks of nude mice. When the average tumor volume reached ~50 mm^3^, mice were divided randomly into two groups (six mice per group) and treated with either vehicle (10% DMSO, 20% cremophor EL and 70% NaCl, i.p.) or chaetocin (0.5 mg/kg, i.p.) every other day for a total of 24 d. Tumors were measured every other day, and tumor volumes were calculated using the following formula: a^2^ × b/2, where a is the smallest diameter and b is the diameter perpendicular to a. At the end of the study, tumors were removed from sacrificed mice and then weighed and stored.

The patient-derived xenograft (PDX) models used in this study were established by our group as described previously^28^. Briefly, fresh gastric cancer tissues, which were termed as F0, were cut into approximately 3 × 3 × 3 mm^3^ pieces and then were subcutaneously injected into the flanks of five-week-old male NOD-Prkdc^em26Cd52^Il2rg^em26Cd22^/Nju (NCG) mice (F1). After tumors reached a size of ~500 mm^3^, they were harvested and passaged into the next generation. One of the successfully established PDX models in the third generation (F3) was used in this study. After 19 d of inoculation, mice were divided randomly into two groups (five mice per group) and treated with either vehicle (10% DMSO, 20% cremophor EL and 70% NaCl, i.p.) or chaetocin (0.25 mg/kg, i.p.) every other day for a total of 14 d. Tumors were measured every other day, and tumor volumes were calculated using the following formula: a^2^ × b/2, where a is the smallest diameter and b is the diameter perpendicular to a. At the end of the study, tumors were removed from sacrificed mice and then weighed and stored.

All animal studies were conducted according to the guidelines of Animal Care and Use Committee of Guangdong Laboratory Animals Monitoring Institute.

### Statistical analysis

All experiments were performed at least 3 times, and the results are presented as the mean ± SD where applicable. Statistical analysis was performed using one-way analysis of variance followed by tukey’s test among multiple groups and Student’s *t*-test between two groups by GraphPad Prism software (San Diego, CA, USA). *P* < 0.05 was considered statistically significant.

## Results

### Chaetocin inhibits the growth of GC cells

**Fig. 1:**
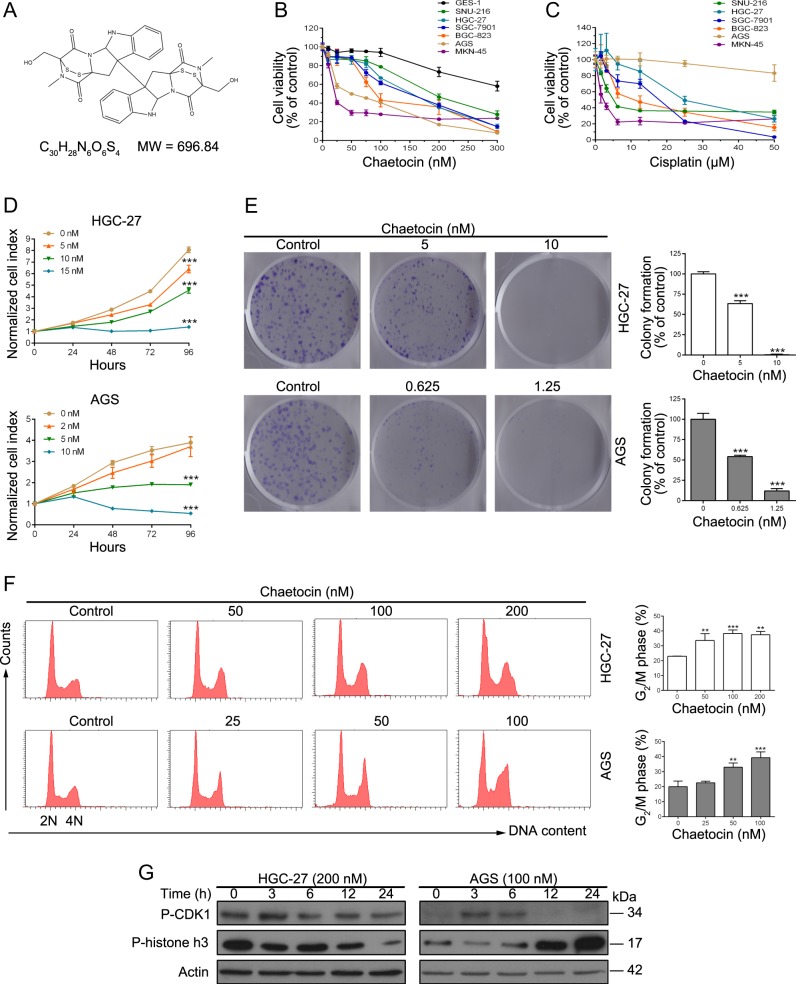
Chaetocin induces G_2_/M phase arrest and inhibits proliferation in GC cells. **a** Chemical structure of chaetocin. **b** and **c** CCK-8 assays were used to measure cell viability following treatment with chaetocin or cisplatin at the indicated concentrations for 24 h. **d** HGC-27 and AGS cells were treated with various concentrations of chaetocin and then cell proliferation was detected by real-time analysis. **e** HGC-27 and AGS cells were incubated with the indicated concentrations of chaetocin for 9 d and then HGC-27 and AGS cell colonies were counted. **f** HGC-27 and AGS cells were exposed to the indicated concentrations of chaetocin for 12 h and then cell cycle distribution was analyzed by flow cytometry. **g** Expression levels of p-CDK1 and p-histone h3 in HGC-27 and AGS were detected by western blot analysis. Blots presented here are representative of three independent experiments. Results in (**b**)–(**f**) are shown as mean ± SD of three independent experiments. **P *<* 0.01, ***P *<* 0.001, vs. control group

The chemical structure of chaetocin is shown in Fig. 1a. We first investigated the cytotoxic effects of chaetocin in GC cells and gastric mucosa epithelial cell line GES-1. Cells were treated with chaetocin for 24 h, and cell viability was detected using a CCK-8 assay. As shown in Fig. 1b and Table 1, GC cell viability was significantly inhibited by very low concentrations of chaetocin (IC_50_ values between 185.7 and 31.0 nM) and GC cells seemed to be more sensitive to chaetocin compared with GES-1. Surprisingly, IC_50_ values of chaetocin to GC cells were lower to those of cisplatin (Fig. 1c and Table 2), which is one of the most effective chemotherapeutic agents used for GC treatment. Two cell lines HGC-27 and AGS, which showed different sensitive to chaetocin, were chosen for further study. To test the effect of chaetocin on GC cell proliferation, real-time cell analysis and colony formation assays were then performed and low concentrations which wouldn’t cause cell death were chosen. Chaetocin reduced both the normalized cell index and colony formation in a dose-dependent manner in HGC-27 and AGS cells (Fig. 1d, e), indicating that GC cell proliferation was markedly restrained. In addition, we used flow cytometry to evaluate the effect of chaetocin on cell cycle distribution. As shown in Fig. 1f, after 12 h of chaetocin treatment, the population of HGC-27 and AGS cells in the G_2_/M phase increased substantially, suggesting that chaetocin induces GC cell arrest in the G_2_/M phase. To determine GC cells were arrested in which phases: G_2_ or mitosis, the expression of p-CDK1 and p-histone h3 proteins were tested; the expression levels of both p-CDK1 and p-histone h3 decrease if cells are arrested in G_2_ phase and the expression levels of p-histone h3 increase during mitosis^29,30^. Interestingly, the expressions of both p-CDK1 and p-histone h3 decreased upon chaetocin treatment in HGC-27 cells while the expression of p-histone h3 increased in AGS cells (Fig. 1g), indicating that chaetocin causes G_2_ arrest in HGC-27 cells while mitotic arrest in AGS cells.

**Table 1:** Effects on viability of chaetocin to GC cells and GES-1

Cell line	IC_50_ (nM)
GES-1	>300
SNU-216	185.7 ± 13.9
HGC-27	141.80 ± 19.6
SGC-7901	133.9 ± 12.8
BGC-823	104.4 ± 4.3
AGS	51.7 ± 7.8
MKN-45	31.0 ± 5.6

**Table 2:** Effects on viability of cisplatin to various GC cells

Cell line	IC_50_ (μM)
SNU-216	7.9 ± 0.6
HGC-27	27.0 ± 3.0
SGC-7901	15.1 ± 3.1
BGC-823	12.2 ± 2.9
AGS	>50
MKN-45	1.5 ± 0.5

### Chaetocin induces caspase-dependent apoptosis in GC cells

**Fig. 2:**
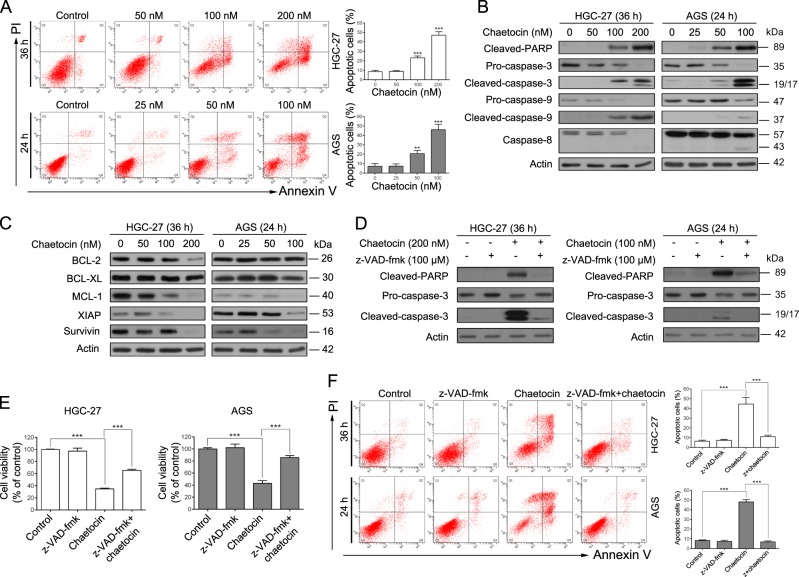
Chaetocin induces caspase-dependent apoptosis in GC cells. **a** Apoptosis of HGC-27 and AGS cells was determined by flow cytometry following treatment with the indicated doses of chaetocin. Results are shown as mean ± SD of three independent experiments. **P *<* 0.01, ***P *<* 0.001, vs. control group. **b** Expression levels of cleaved-PARP, caspase-3, caspase-9 and caspase-8 in HGC-27 and AGS cells were analyzed by western blot. **c** Expression levels of BCL-2, BCL-XL, MCL-1, XIAP and survivin in HGC-27 and AGS cells were analyzed by western blot. HGC-27 and AGS cells were pretreated with 100 μM pan-caspase inhibitor z-VAD-fmk for 2 h and then cotreated with chaetocin (200 nM for HGC-27, 100 nM for AGS). **d** Expression levels of cleaved-PARP and caspase-3 were detected by western blot. **e** Cell viability was measured by CCK-8 assays. **f** Apoptosis was analyzed by flow cytometry. Results in (**e**) and (**f**) are shown as mean ± SD of three independent experiments. ***P *<* 0.001. All blots presented here are representative of three independent experiments

We next determined whether chaetocin could induce apoptotic cell death in GC cells. HGC-27 and AGS cells were exposed to chaetocin and then assessed by flow cytometry with annexin V-FITC/PI staining. Apoptotic HGC-27 and AGS cell populations increased significantly following chaetocin treatment (Fig. 2a). To further confirm the chaetocin-induced apoptotic effect, apoptosis-related proteins were detected by western blot assay. As shown in Fig. 2b, chaetocin-induced PARP cleavage (an indicator of apoptosis) in HGC-27 and AGS cells. Further, caspase family proteins (known upstream activators of PARP) were activated. Chaetocin decreased levels of the precursor forms of caspase-3, −9, and −8 and increased the cleaved forms of caspase-3, −9, and −8 in HGC-27 and AGS cells, illustrating that chaetocin may induce caspase-dependent apoptosis of GC cells. Finally, we measured the expression of BCL-2 and IAP family proteins, as these proteins can affect apoptosis rates. Western blot analysis demonstrated that levels of the anti-apoptotic proteins BCL-2, BCL-XL, MCL-1, XIAP and survivin decreased following chaetocin treatment (Fig. 2c). Taken together, these results showed that chaetocin induces GC cell apoptosis. To verify whether the chaetocin-induced GC cell apoptosis was caspase-dependent, we treated cells with the pan-caspase inhibitor z-VAD-fmk and then assessed chaetocin-induced apoptotic effects. z-VAD-fmk significantly blocked chaetocin-induced caspase pathway activation (Fig. 2d). Figure 2e, f showed that chaetocin-induced viability inhibition and apoptotic effect was significantly reversed by z-VAD-fmk treatment in HGC-27 and AGS cells, indicating that chaetocin induces apoptosis in GC cells through the caspase pathway.

### Chaetocin induces GC cell apoptosis by inhibiting TRXR-1 activity

**Fig. 3:**
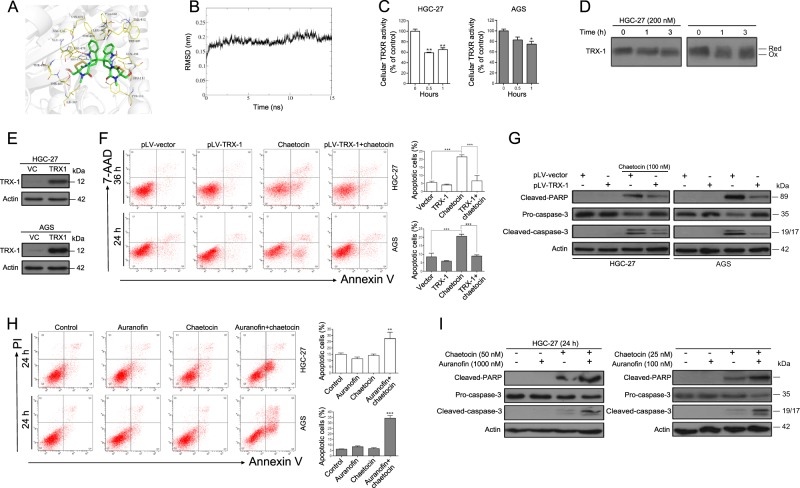
Chaetocin-inactivated TRXR-1 is required for GC cell apoptosis. **a** Molecular docking between chaetocin and TRXR-1 was simulated using the AutoDock 4.2 program. **b** RMSD trajectories of chaetocin and TRXR-1 in a 15 ns MD simulation. **c** TRXR-1 activity was determined by DTNB assay in HGC-27 and AGS cells treated with 200 nM and 100 nM chaetocin, respectively, for the indicated lengths of time. Results are shown as mean ± SD of three independent experiments. *P *<* 0.05, **P *<* 0.01, vs. control group. **d** Lysates of HGC-27 and AGS cells treated with 200 nM and 100 nM chaetocin, respectively, for the indicated lengths of time were alkylated by AMS and the redox state of TRX-1 was separated by non-reducing SDS-PAGE followed by western blot analysis. Reduced and oxidized forms are indicated. **e** TRX-1 expression levels in TRX-1-overexpressing HGC-27 and AGS cells were determined by western blot. TRX-1-overexpressing HGC-27 and AGS cells were treated with chaetocin (100 nM for HGC-27, 50 nM for AGS). **f** Apoptosis was analyzed by flow cytometry. Results were shown as mean ± SD of three independent experiments. ***P *<* 0.001. **g** The expression levels of cleaved-PARP and caspase-3 were detected by western blot. HGC-27 and AGS cells were cotreated with auranofin (1000 nM for HGC-27, 100 nM for AGS) and chaetocin (50 nM for HGC-27, 25 nM for AGS). **h** Apoptosis was analyzed by flow cytometry. Results were shown as mean ± SD of three independent experiments. **P *<* 0.01, ***P *<* 0.001, vs. control group. **i** The expression levels of cleaved-PARP and caspase-3 were detected by western blot. All blots presented here are representative of three independent experiments

As the TRXR-1-inhibitory effects of chaetocin have already been described in cell-free assays^25^, we first performed molecular docking studies to further confirm the chaetocin-TRXR-1 interaction and to investigate possible binding sites. The docking results showed that chaetocin could bind TRXR-1 directly with a binding energy as high as −9.5 kcal/mol. The binding interactions consisted of a hydrogen bond between chaetocin and either the Tyr116 or the Glu477 residue in TRXR-1 (Fig. 3a). As the molecular docking method indicated a possibly instantaneous binding mode, we then used MD simulations to evaluate the reasonable/stable binding patterns between chaetocin and TRXR-1. As shown in Fig. 3b, the root mean square deviation (RMSD) curve was stable at approximately 1.8–2.2 Å after 2 ns, implying a powerful binding interaction between chaetocin and TRXR-1. To calculate the binding free energy and speculate upon the possible residues involved in chaetocin binding, we used MM/PBSA to investigate the detailed changes in 200 frames within the final 5 ns. The binding free energy between chaetocin and TRXR-1 is −157.013 kJ/mol and the top 10 possible binding residues are shown in Table 3. Consistent with the docking results, chaetocin can form two hydrogen bonds with Glu477. We next tested the inhibitory effects of chaetocin on TRXR-1 activity in GC cells. Following treatment of HGC-27 and AGS cells with chaetocin, the activity of TRXR-1 was significantly inhibited (Fig. 3c). And this effect is similar to auranofin, which has been previously reported to induce GC cell death by inhibiting the activity of TRXR-1 followed by inducing ROS accumulation^31,32^ (Supplementary Figs. 1–3, Supplementary Table 1). TRXR-1 provides reducing equivalents to the downstream effector TRX-1, which is ultimately responsible for the antioxidant effects^5^. The inhibition of TRXR-1 activity induced by chaetocin might lead to a decrease of reduced TRX-1. We then investigated the redox state of TRX-1 in GC cells by using AMS alkylation method. As shown in Fig. 3d, the amount of oxidized TRX-1 increased when GC cells were treated with chaetocin. Taken together, these results demonstrate that chaetocin can inactivate TRXR-1 in GC cells.

**Table 3:** Top 10 possible binding residues in TRXR-1 with chaetocin

Residues	MM energy (kJ/mol)
Glu-477	−33.69
Gln-494	−21.56
Thr-481	−7.78
Val-478	−7.68
Tyr-116	−7.24
Glu-122	−6.04
Gly-499	−5.86
Pro-344	−5.41
His-472	−5.41
Thr-480	−4.75

As chaetocin-induced inhibition of TRXR-1 may result in a reduction of reduced TRX-1, and finally induce cell apoptosis via accumulation of ROS. We therefore hypothesized that increased TRX-1 could diminish chaetocin-induced apoptosis. We constructed cells that stably overexpressed TRX-1 (Fig. 3e) and found that stable TRX-1 overexpression in HGC-27 and AGS cells attenuated chaetocin-induced apoptosis (Fig. 3f, g). We next investigated whether chaetocin-induced apoptosis was increased when cells were cotreated with auranofin, a reported inhibitor of thioredoxin reductase. The combined treatment induced much higher apoptosis in GC cells than that of single agent (Fig. 3h, i). These results suggest that chaetocin induces GC cell apoptosis by inhibiting TRXR-1 activity.

### Chaetocin-induced GC cell apoptosis depends on ROS generation

**Fig. 4:**
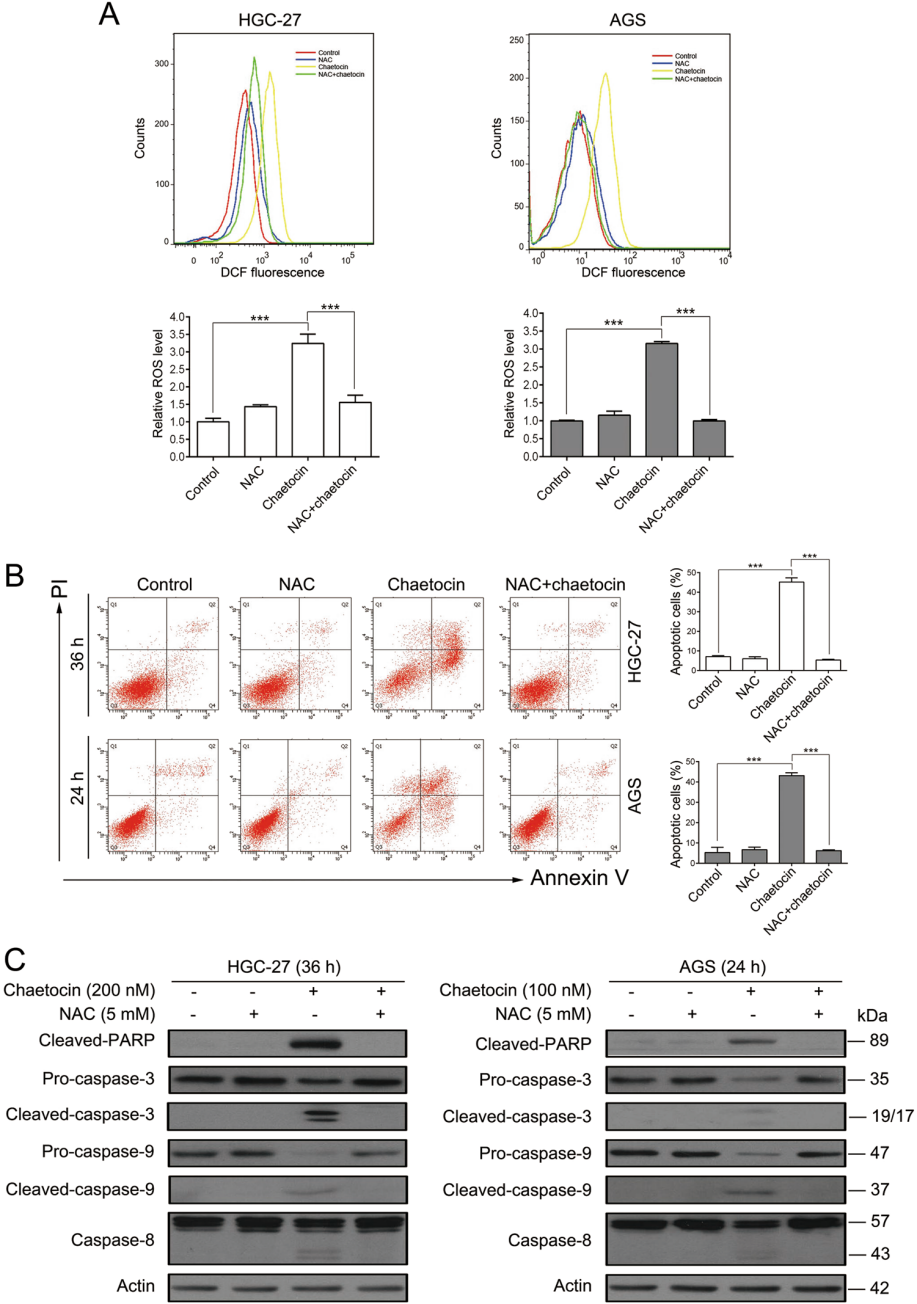
Chaetocin-induced accumulation of ROS is crucial for GC cell apoptosis. **a** ROS levels were analyzed by flow cytometry in HGC-27 and AGS cells treated for 1 h with 5 mM NAC, chaetocin (200 nM for HGC-27, 100 nM for AGS), or both. Results were shown as mean ± SD of three independent experiments. ***P *<* 0.001. HGC-27 and AGS cells were pretreated with 5 mM NAC for 1 h and then cotreated with chaetocin (200 nM for HGC-27, 100 nM for AGS). **b** Apoptosis was measured by flow cytometry. Results were shown as mean ± SD of three independent experiments. ***P *<* 0.001. **c** Expression levels of cleaved-PARP, caspase-3, caspase-9 and caspase-8 were analyzed by western blot. Blots presented here are representative of three independent experiments

Because the TRX-TRXR system acts as a ROS scavenger, we next determined ROS levels in chaetocin-treated GC cells. ROS levels in GC cells, detected using a fluorescent DCFH/DA probe, increased significantly upon chaetocin treatment (Fig. 4a). Previous studies have demonstrated that increased ROS production is crucial for chaetocin-induced apoptotic cell death in leukemia, glioma, and myeloma cells^18,33,34^. Therefore, we next determined the role of ROS in the chaetocin-induced apoptosis of GC cells. We used NAC to inhibit the production of ROS (Fig. 4a). Flow cytometry analysis demonstrated that the chaetocin-induced increase in apoptotic cells was almost completely reversed upon cotreatment with NAC (Fig. 4b). In accordance with the flow cytometric results, western blot demonstrated that NAC treatment could reverse the chaetocin-induced increase in cleaved PARP and caspase family proteins (Fig. 4c). These results suggest that chaetocin induces ROS production by inhibiting the TRX-TRXR system and that this ROS production is required for chaetocin-mediated apoptosis in GC cells.

### The PI3K/AKT pathway is involved in chaetocin-induced GC cell apoptosis

**Fig. 5:**
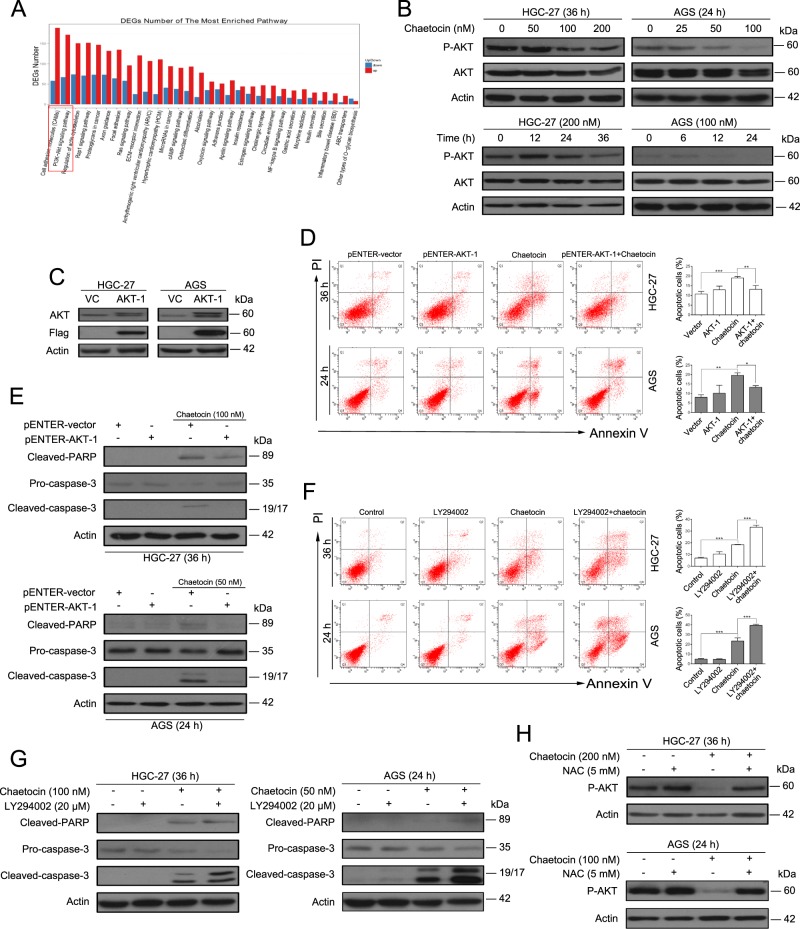
The PI3K/AKT pathway is involved in chaetocin-induced apoptosis in GC cells. **a** RNA-seq was performed on non-treated AGS cells as well as AGS cells with 100 nM chaetocin treatment for 12 h. And DEGs defined as +/− 2-fold change and FDR < 0.01 were included for enriched gene pathway analysis. **b** p-AKT and AKT expression levels were analyzed by western blot in chaetocin-treated HGC-27 and AGS cells. **c** AKT and flag-tag expression levels were detected by western blot in HGC-27 and AGS cells transiently overexpressing AKT. AKT-overexpressing HGC-27 and AGS cells were treated with chaetocin (100 nM for HGC-27, 50 nM for AGS). **d** Apoptosis was analyzed by flow cytometry. Results were shown as mean ± SD of three independent experiments. *P *<* 0.05, **P *<* 0.01, ***P *<* 0.001. **e** Cleaved-PARP and caspase-3 expression levels were determined by western blot. HGC-27 and AGS cells were pretreated with 20 μM LY294002 for 2 h and then cotreated with chaetocin (100 nM for HGC-27, 50 nM for AGS). (**f**) Apoptosis was analyzed by flow cytometry. Results were shown as mean ± SD of three independent experiments. ***P *<* 0.001. **g** Cleaved-PARP and caspase-3 expression levels were determined by western blot. (**h**) HGC-27 and AGS cells were pretreated with 5 mM NAC for 1 h and then cotreated with chaetocin (200 nM for HGC-27, 100 nM for AGS). Then, p-AKT expression levels were analyzed by western blot. All blots presented here are representative of three independent experiments

To demonstrate the detailed mechanism underlying TRXR-1/TRX-1/ROS-mediated apoptosis, we performed RNA-seq analysis of chaetocin-treated AGS cells. Several pathways were affected by chaetocin treatment; one of the most enriched pathways was the PI3K/AKT pathway (Fig. 5a). The PI3K/AKT pathway is one of the most well-known ROS-regulated pathways and plays an important role in cancer progression by promoting cancer cell proliferation and inhibiting cancer cell apoptosis^35,36^. Thus, we focused on the role of the PI3K/AKT pathway in chaetocin-mediated cell death. We first verified the effect of chaetocin on the PI3K/AKT pathway in HGC-27 and AGS cells. The expression of some genes, like c-Myc, Axin-2, BCL-XL, MCL-1 and XIAP, may be downregulated if the PI3K/AKT pathway is inactivated^37^. Our results revealed that the mRNA expression levels of c-Myc, Axin-2, BCL-XL, MCL-1, and XIAP were downregulated in GC cells treated with chaetocin (Supplementary Fig. 4), which is consistent with the RNA-seq analysis. Moreover, following chaetocin treatment, phospho-AKT (Ser473) expression decreased in a dose- and time-dependent manner, while total AKT expression remained nearly unchanged (Fig. 5b). These results indicate that the PI3K/AKT pathway is inactivated by chaetocin in GC cells. To further confirm that the PI3K/AKT pathway is involved in chaetocin-induced cell death, we transiently overexpressed AKT-1 in GC cells and then observed the effect of chaetocin on these cells (Fig. 5c). Both flow cytometry and western blot demonstrated that AKT-1 overexpression could slightly attenuate chaetocin-induced apoptosis (Fig. 5d, e). Moreover, the PI3K/AKT pathway inhibitor LY294002 significantly enhanced chaetocin-induced apoptotic effects (Fig. 5f, g). Studies have reported that ROS can repress the PI3K/AKT pathway^38^. Our results revealed that the ROS scavenger NAC could rescue the chaetocin-induced decrease in p-AKT levels (Fig. 5h). These data indicate that the chaetocin-mediated inactivation of the PI3K/AKT pathway depends on ROS generation and is involved in chaetocin-induced cell apoptosis.

### Chaetocin inhibits the growth of GC cell xenografts and patient-derived GC xenografts

**Fig. 6:**
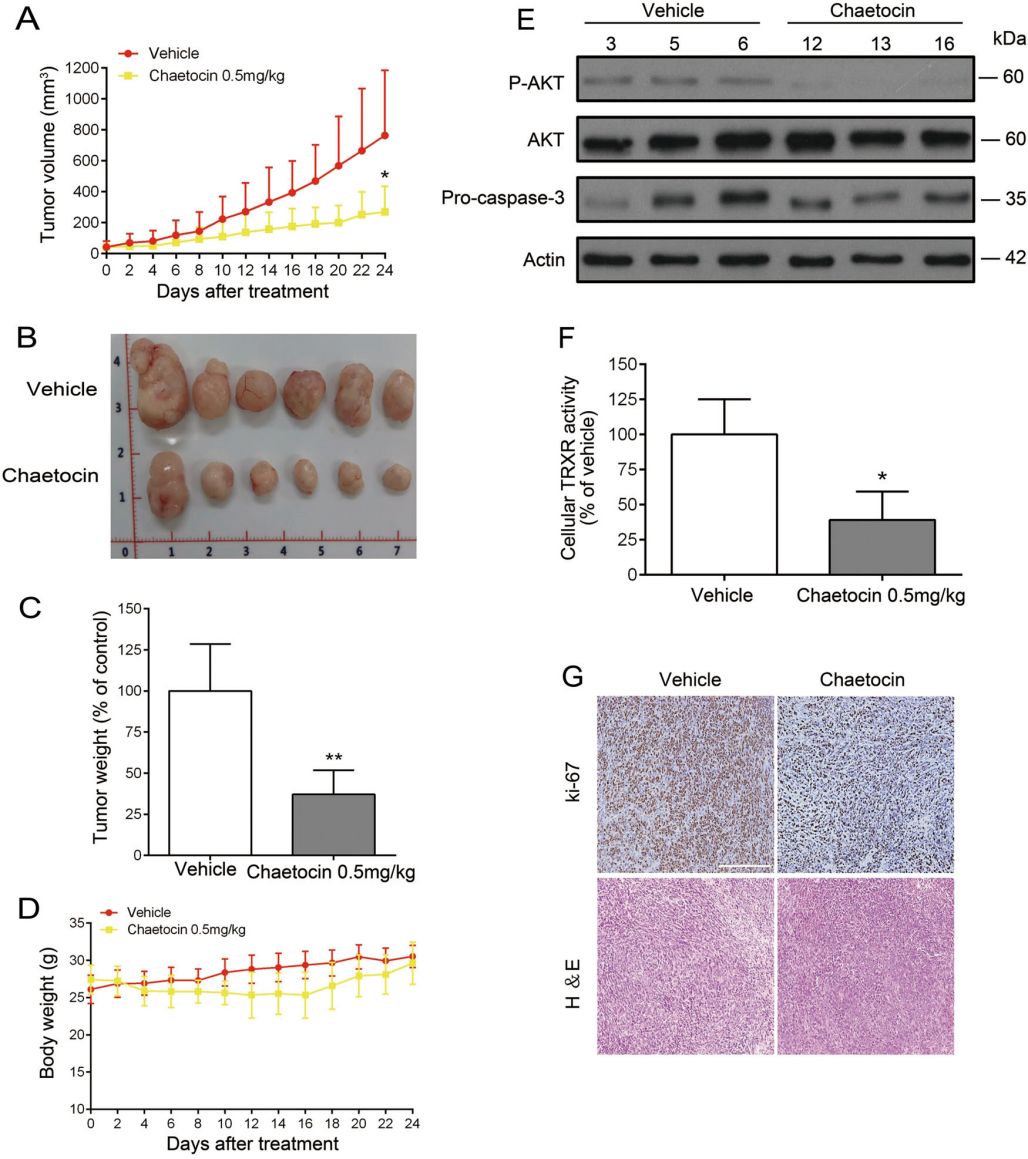
Chaetocin inhibits the growth of HGC-27 cell xenografts. Nude mice were subcutaneously inoculated with HGC-27 and then treated with vehicle or chaetocin (0.5 mg/kg) for 24 d. **a** and **b** tumor volume, and **c** tumor weight of HGC-27 GC cell xenografts in nude mice. **d** Body weight of the mice. **e** Levels of p-AKT, AKT and pro-caspase-3 in tumor tissues were detected by western blot (HGC-27 vehicle group: 3, 5, 6; HGC-27 chaetocin-treated group: 12, 13, 16). **f** TRXR activity in tumor tissues was determined by DTNB assay. **g** Ki-67 expression in tumor tissues was detected using immunohistochemistry. Scale bar: 100 μm. Results were shown as mean ± SD of six mice in each group. *P *<* 0.05, **P *<* 0.01, vs. control group

The effect of chaetocin on GC cells in vivo was examined using a nude mouse xenograft model. HGC-27 cells were subcutaneously implanted into nude mice. Then, mice were treated with either vehicle or chaetocin (0.5 mg/kg, i.p.) every other day for 24 d. Compared with vehicle treatment, chaetocin effectively reduced the volume and weight of HGC-27 xenograft tumors (Fig. 6a–c), and no significant losses were detected in the body weights of the experimental animals (Fig. 6d), suggesting that chaetocin significantly restrains the growth of GC cell xenografts but has no major side effects in mice. Consistent with our in vitro results, chaetocin-treated tumor tissues displayed decreased pro-caspase-3 and p-AKT protein levels and attenuated TRXR activity (Fig. 6e, f). Moreover, immunohistochemistry staining demonstrated that the expression of ki-67, a biomarker related to proliferation, was downregulated in response to chaetocin (Fig. 6g).

**Fig. 7:**
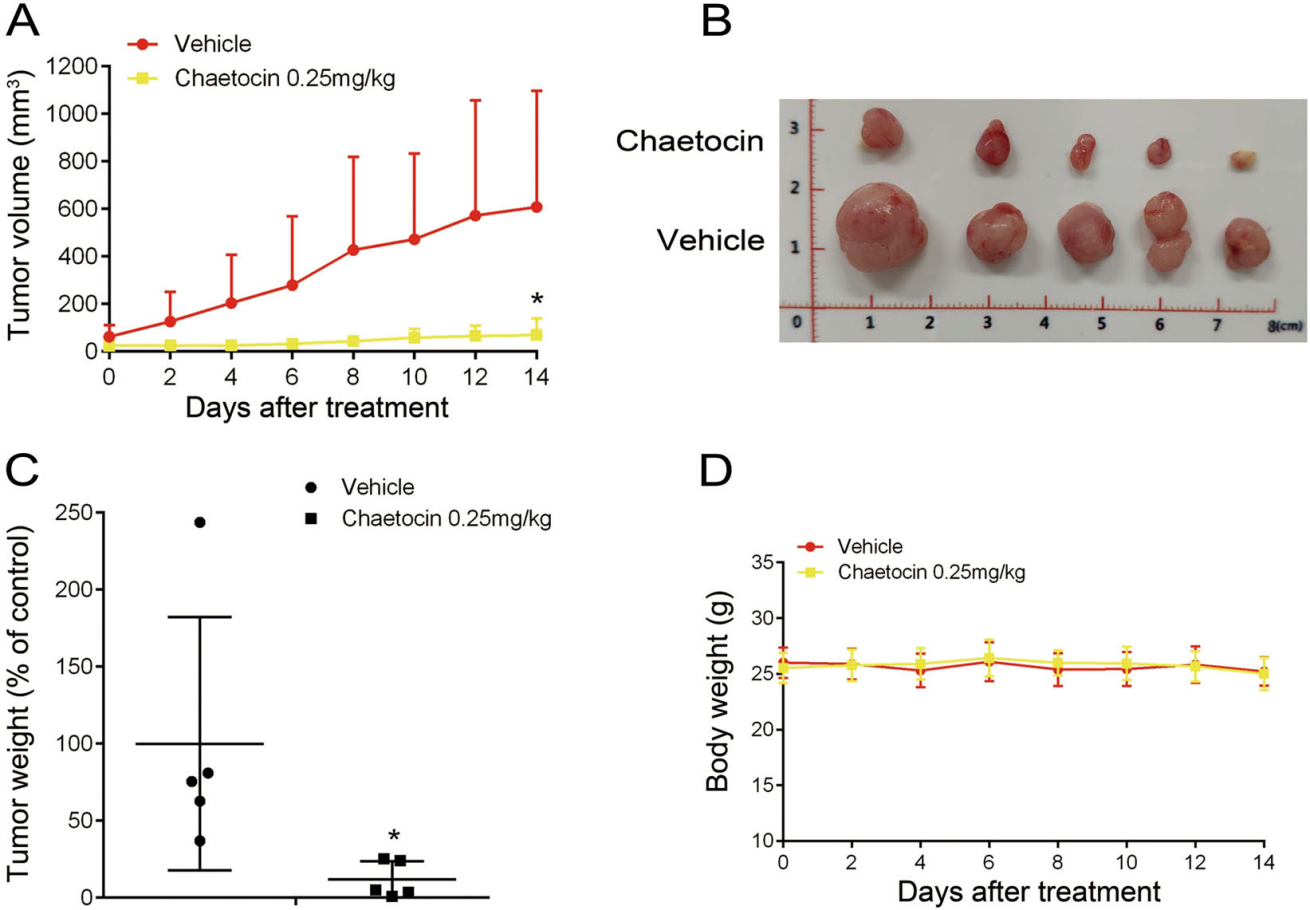
Chaetocin inhibits the growth of patient-derived GC xenografts. NCG mice were subcutaneously inoculated with GC tissues and then treated with vehicle or chaetocin (0.25 mg/kg) for 14 d. **a** and **b** tumor volume, and **c** tumor weight of patient-derived xenografts in NCG mice. **d** Body weight of the mice. Results were shown as mean ± SD of five mice in each group. *P *<* 0.05, vs. control group

PDX animal models maintain the heterogeneity of human cancers and can accurately predict the responses of clinical drugs or novel agents in cancer treatment. We then detected the effect of chaetocin on patient-derived GC xenografts. Aligned with the results from the GC cell xenografts, the volume and weight of PDX were significantly deceased in chaetocin-treated group Compared with the vehicle group and no significant changes in mice body weights were found in both groups (Fig. 7a–d).

Taken together, these data indicate that chaetocin inhibits tumor growth in vivo by targeting TRXR-1.

## Discussion

GC is one of the most common and most lethal cancers worldwide^1^. Although traditional chemotherapeutic drugs such as cisplatin and new molecular targeted agents such as trastuzumab can improve the survival rates of GC patients, the curative effects of these drugs are still not satisfactory^2,3^. Therefore, novel therapeutic agents need to be developed for the treatment of GC. In the present study, we determined that low concentrations of chaetocin could induce growth inhibition and caspase-dependent apoptosis in GC cells both in vitro and in vivo through the inhibition of TRXR-1 and the subsequent accumulation of ROS. Moreover, the ROS-dependent inactivation of the PI3K/AKT pathway was involved in chaetocin-induced cell apoptosis. To our knowledge, this is the first report to demonstrate that chaetocin inhibits the proliferation and induces apoptosis of GC cells in vitro and in vivo, which suggests that chaetocin may be a potential candidate for GC treatment.

ROS play important roles in cell growth, differentiation and death through the modification of various signaling molecules^39–41^. It has been demonstrated that ROS are implicated in diverse diseases, including cancers. Compared with their normal counterparts, cancer cells display inherently elevated ROS levels. Increased ROS levels are closely related to cancer initiation, metastasis, and drug resistance^42,43^. Although moderate increases in ROS levels can be beneficial to cancer cells, excessive amounts of ROS can cause cell death^44,45^. Therefore, by modulating ROS levels, redox-targeted agents may effectively kill cancer cells. In fact, some small molecules (for example, cardamonin and furanodienone) have been shown to effectively eliminate cancer cells by increasing ROS levels in cancer cells^46,47^. By upregulating the antioxidant system, cancer cells hinder excessive ROS accumulation and prevent ROS buildup beyond lethal levels. One of the most important antioxidant systems is the TRX-TRXR system^48^. Interestingly, the TRX-TRXR system has been found to be hyperactivated in many cancers including gastric, colorectal and lung cancer. Further, hyperactivation of the TRX-TRXR system plays a critical role in tumorigenesis and cancer progression^8–11,49^. Therefore, the TRX-TRXR system may be a valuable drug target for cancer therapy. Indeed, some agents such as cisplatin^50,51^, one of the most common chemotherapeutic agents used to treat GC, can effectively eliminate cancer cells by inhibiting the TRX-TRXR system. Although cisplatin has been shown to inhibit the viability of GC cells by targeting TRXR-1, the curative effects of cisplatin are still not satisfactory. Therefore, new and more effective agents need to be developed.

Recently, chaetocin, a small-molecule natural product produced by the *Chaetomium* species of fungi, has been shown to have strong cytotoxic effects against human cancer cells^15,19^. The cytotoxic effects of chaetocin against cancer cells may be related to TRXR-1 inhibition^25,34^. However, the pharmacological mechanism of action of chaetocin in GC has not yet been reported. Our results showed that very low concentrations of chaetocin can inhibit growth and induce apoptotic cell death in GC cells (Figs. 1, 2). We further demonstrated that chaetocin increased cellular ROS production via inhibition of TRXR-1 in GC cells (Figs. 3c and 4a). Moreover, our results showed that overexpression of TRX-1 or cotreatment with NAC could attenuate the cytotoxic effects of chaetocin in GC cells (Figs. 3f, g and 4b, c). These results present convincing evidence that chaetocin is an effective antiGC agent and that its effect may depend on the inhibition of TRXR-1 and the subsequent accumulation of excessive ROS levels.

Human TRXR-1 contains two identical subunits arranged head-to-tail. A Cys59-Cys64 active site pair in one subunit receives electrons from NADPH and transfers them to the Cys497-selenoCys498 pair at the C-terminus of the other subunit; in this manner, TRXR-1 is maintained in its reduced form and can function as an active enzyme^52,53^. Tibodeau et al. found that chaetocicn inhibited the activity of TRXR-1 in the cell-free system and postulated that chaetocin might interact with the Cys497-selenoCys498 pair at the C-terminus according to the above theories^25^. Our results showed that chaetocin binds to TRXR-1 and inhibits its activity in GC cells. However, our molecular docking and MD simulation results indicated that chaetocin primarily interacts with the Glu-477 residue through the formation of hydrogen bonds (Fig. 3a, b). As the structure of TRXR-1 in cells changes dynamically, our simulation results may not accurately depict the final binding mode between chaetocin and the active pocket of TRXR-1. Therefore, in the future, bioengineering techniques should be utilized to determine the exact binding sites of chaetocin with TRXR-1 in GC cells.

It has been reported that chaetocin may exhibit its anticancer activity by interacting with other targets: lysine 9 on histone H3 methyltransferase SUV39H1and HIF-1α^23,24^. Some recent findings have demonstrated that chaetocin is a nonspecific inhibitor of SUV39H1^54^. Further, our results showed that the ROS scavenger NAC partly rescued chaetocin-induced inhibition of trimethylation of lysine 9 on histone H3 (H3K9me3), indicating that SUV39H1 may be one of the downstream molecules involved in the ROS pathway (Supplementary Fig. 5)^33,34^. Isham et al. found that the anticancer effects of chaetocin did not depend on functional HIF-1α^19^. Of note, the anticancer mechanism of chaetocin may vary across different cancers. And the ROS scavenger NAC was able to nearly completely abrogate the anticancer activity of chaetocin in GC cells (Fig. 4b, c), implying that the cytotoxic effects of chaetocin in GC cells may be primarily due to its inhibition of TRXR-1 and the subsequent excessive ROS accumulation, at least in our in vitro model system.

Deregulation of the PI3K/AKT pathway has been reported in various types of cancers, including GC. Additionally, the PI3K/AKT pathway plays an important role in cancer cell proliferation, metastasis, and drug resistance^36^. It is widely known that the PI3K/AKT pathway is a terrific potential molecular target for cancer therapies. Previous studies have shown that ROS can repress the PI3K/AKT pathway^38,55^. However, the relationship between ROS and the PI3K/AKT pathway following chaetocin treatment has not been illustrated yet. Using RNA-seq analysis, we found that the PI3K/AKT pathway is one of the most enriched pathways in chaetocin-treated GC cells (Fig. 5a). Our western blot results confirmed that chaetocin inactivates the PI3K/AKT pathway in GC cells (Fig. 5b). The chaetocin-induced apoptotic cell death could be partially rescued by ectopic expression of constitutively active AKT (Fig. 5d, e). Consistently, the PI3K/AKT-specific inhibitor LY294002 enhanced chaetocin-induced cell apoptosis (Fig. 5f, g). Moreover, the expression of some antiapoptotic proteins (BCL-XL and MCL-1 of the BCL-2 family and XIAP of the IAP family) are regulated by the PI3K/AKT pathway^37^. Our results showed that both mRNA and protein levels of BCL-XL, MCL-1, and XIAP were downregulated in GC cells upon chaetocin treatment (Fig. 2c and Supplementary Fig. 4), indicating that the inhibition of the PI3K/AKT pathway by chaetocin may result in the downregulation of BCL-XL, MCL-1, and XIAP. These results suggest that the PI3K/AKT pathway is involved in chaetocin-induced GC cell apoptosis. As the ectopic expression of AKT could only partially rescue cells from apoptosis, there likely exist other pathways that are involved in chaetocin-induced cell death. Interestingly, when GC cells were cotreated with the ROS scavenger NAC, chaetocin-induced downregulation of p-AKT was restored (Fig. 5h), suggesting that the chaetocin-induced inactivation of AKT is regulated by ROS. Previous studies have shown that ROS can directly oxidize and dephosphorylate AKT, resulting in its inactivation^38^. However, no significant amount of oxidized AKT increased in GC cells treated with chaetocin, indicating chaetocin-induced ROS may not oxidize and inactivate AKT directly (Supplementary Fig. 6). Therefore, a more detailed investigation should be performed to determine the underlying mechanism of ROS-mediated regulation of the PI3K/AKT pathway in chaetocin-treated GC cells.

**Fig. 8:**
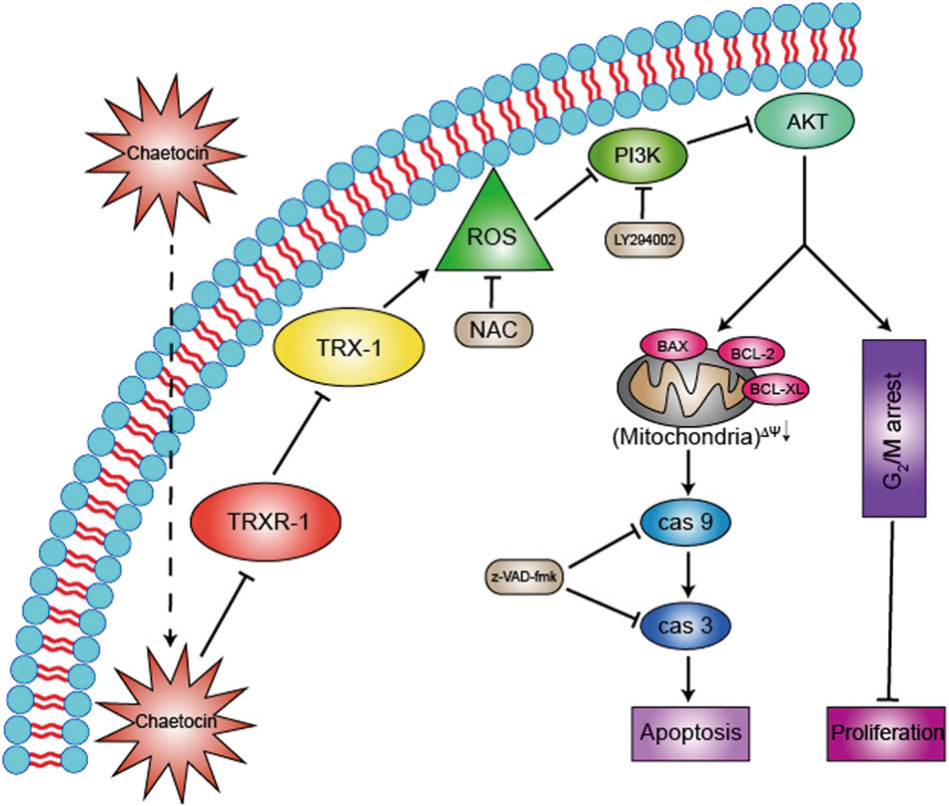
The schematic diagram of possible molecular mechanism of chaetocin-induced cytotoxicity in GC

In summary, our data demonstrate that chaetocin inhibits TRXR-1 and subsequently induces excessive ROS accumulation followed by inactivation of the PI3K/AKT pathway, ultimately leading to GC cell death (Fig. 8). This suggests that chaetocin may be a potential candidate for anti-GC treatment.

## Supplementary information

Supplementary Figure legends

Supplementary Table 1

Supplementary Fig.1

Supplementary Fig.2

Supplementary Fig.3

Supplementary Fig.4

Supplementary Fig.5

Supplementary Fig.6
