# Supplementary material for: ROS-mediated inactivation of the PI3K/AKT pathway is involved in the antigastric cancer effects of thioredoxin reductase-1 inhibitor chaetocin
Source: Cell Death Dis. 2019 Oct 24;10(11):809. doi: 10.1038/s41419-019-2035-x (PMC6813365; doi:10.1038/s41419-019-2035-x)
Supplement: Supplementary file 1 — Supplementary Figure legends [file 41419_2019_2035_MOESM1_ESM.docx]

**Supplementary Figure legends**

**Supplementary Fig.1.** CCK-8 assays were used to determine GC cell viability following treatment with auranofin at the indicated concentrations for 24 h.

**Supplementary Fig.2.** DTNB assay was used to determine the TRXR-1 activity in HGC-27 and AGS cells treated with auranofin for 1 h. Results are shown as mean ± SD of three independent experiments. ^***^*P* < 0.001, versus control group.

**Supplementary Fig.3.** ROS levels were analyzed by flow cytometry in HGC-27 and AGS cells treated with auranofin for 1 h. Results were shown as mean ± SD of three independent experiments. ^*^*P* < 0.05, ^**^*P* < 0.01.

**Supplementary Fig.4.** mRNA expression levels of differentially expressed genes, which were from RNA-seq results and related with the PI3K/AKT pathway, were validated using qpcr in AGS cells treated with chaetocin for 12 h. ^*^*P* < 0.05, ^**^*P* < 0.01 and ^***^*P* < 0.001, versus control group.

**Supplementary Fig.5.** HGC-27 and AGS cells were pretreated with 5 mM NAC for 1 h and then cotreated with chaetocin. Expression levels of trimethylation of lysine 9 on histone H3 were analyzed by western blot. Blots presented here are representative of three independent experiments.

**Supplementary Fig.6.** Lysates of HGC-27 and AGS cells treated with 200 nM and 100 nM chaetocin, respectively, for the indicated lengths of time were alkylated by AMS and the redox state of AKT was separated by non-reducing SDS-PAGE followed by western blot analysis.
