# Supplementary material for: ROS-mediated inactivation of the PI3K/AKT pathway is involved in the antigastric cancer effects of thioredoxin reductase-1 inhibitor chaetocin
Source: Cell Death Dis. 2019 Oct 24;10(11):809. doi: 10.1038/s41419-019-2035-x (PMC6813365; doi:10.1038/s41419-019-2035-x)
Supplement: Supplementary file 2 — Supplementary Table 1 [file 41419_2019_2035_MOESM2_ESM.docx]

| Cell line | IC_50_ (μM) |
| --- | --- |
| SNU-216  HGC-27  SGC-7901  BGC-823  AGS  MKN-45 | 2.15 ± 0.32  1.03 ± 0.18  1.55 ± 0.09  1.80 ± 0.08  0.36 ± 0.03  3.06 ± 0.12 |

**Supplementary Table 1** Effects on viability of auranofin to various GC cells
