# Supplementary figures and images for: ROS-mediated inactivation of the PI3K/AKT pathway is involved in the antigastric cancer effects of thioredoxin reductase-1 inhibitor chaetocin
Source: Cell Death Dis. 2019 Oct 24;10(11):809. doi: 10.1038/s41419-019-2035-x (PMC6813365; doi:10.1038/s41419-019-2035-x)

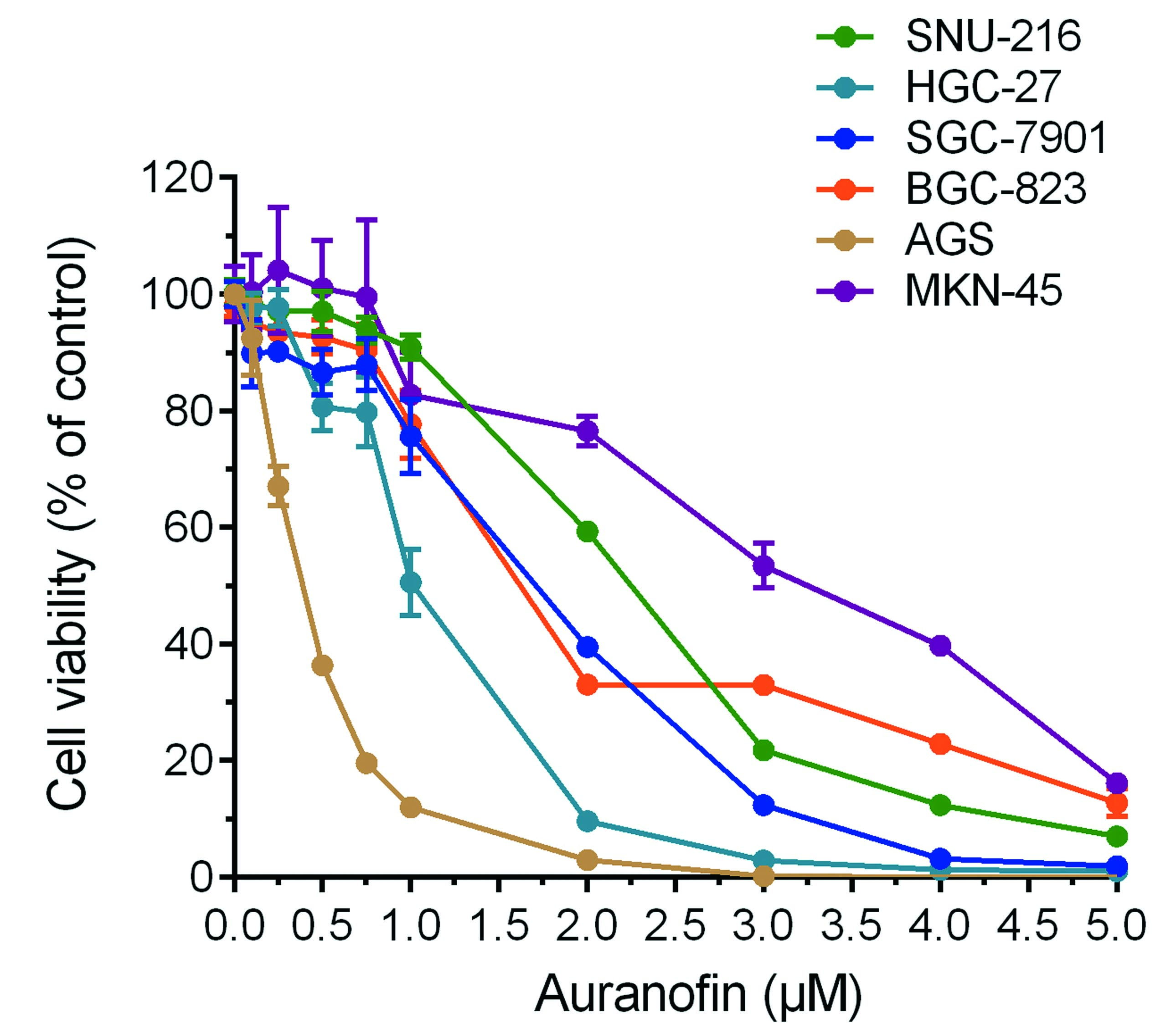

Supplement: Supplementary file 3 — Supplementary Fig.1 [file 41419_2019_2035_MOESM3_ESM.jpg]

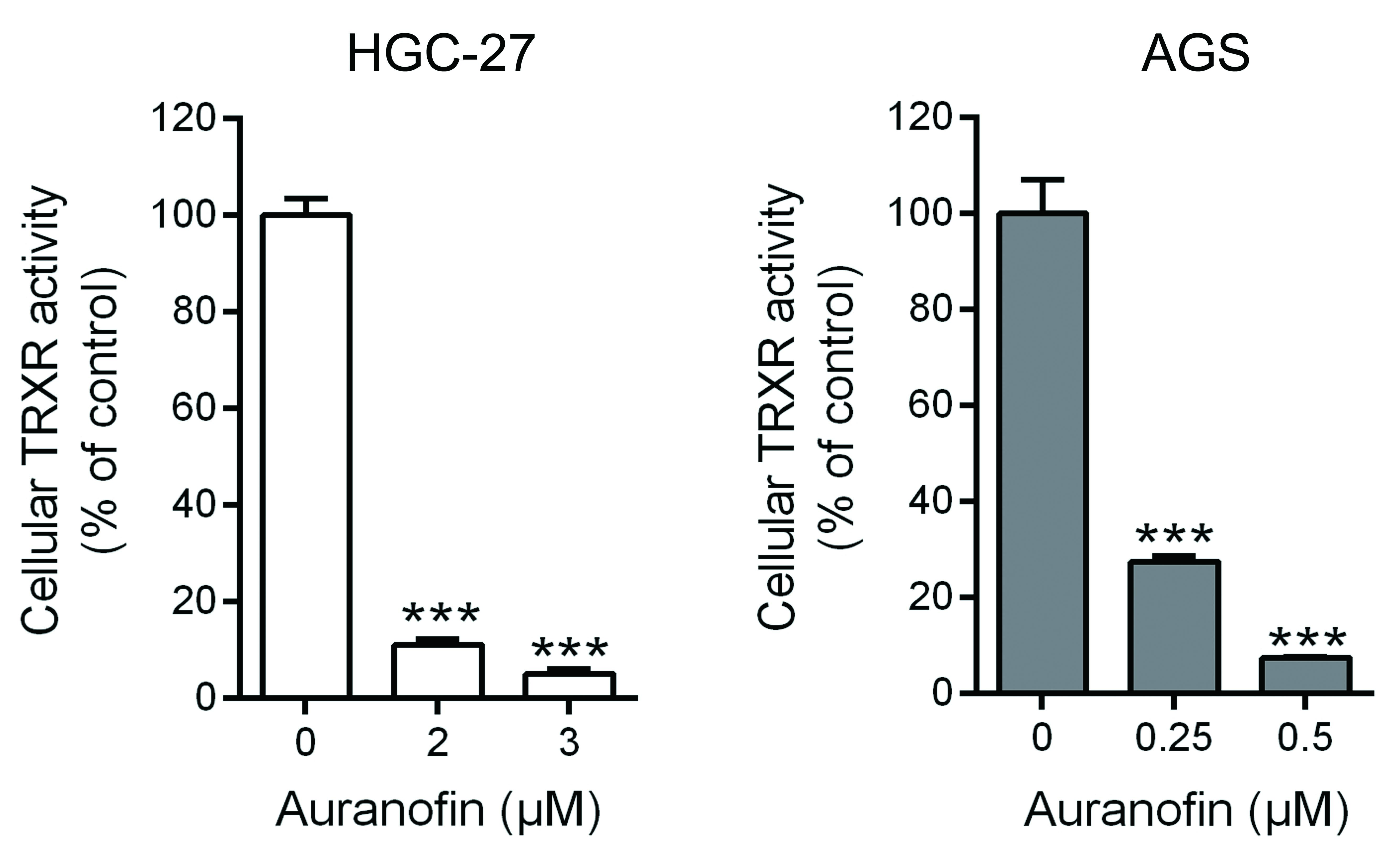

Supplement: Supplementary file 4 — Supplementary Fig.2 [file 41419_2019_2035_MOESM4_ESM.jpg]

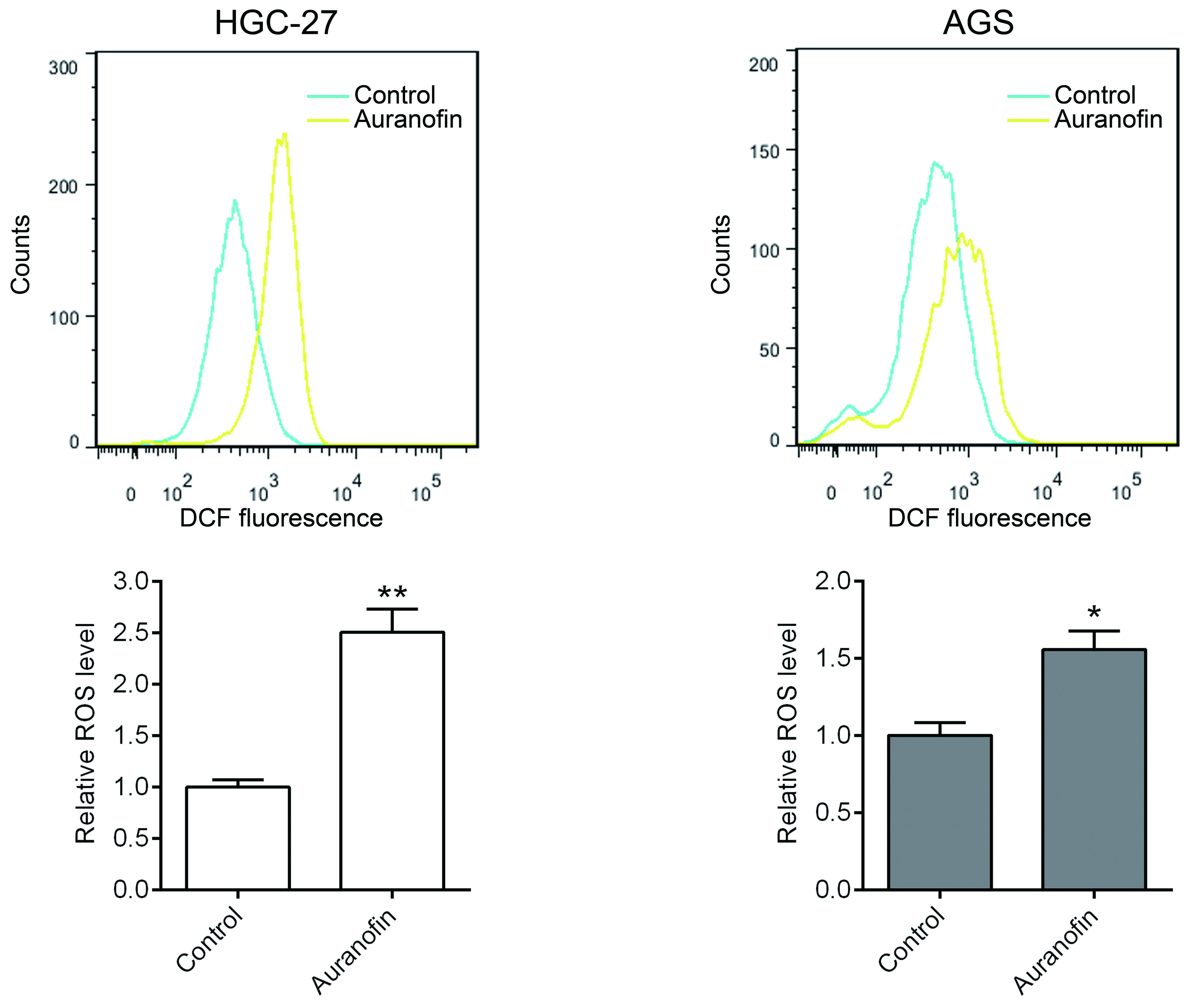

Supplement: Supplementary file 5 — Supplementary Fig.3 [file 41419_2019_2035_MOESM5_ESM.jpg]

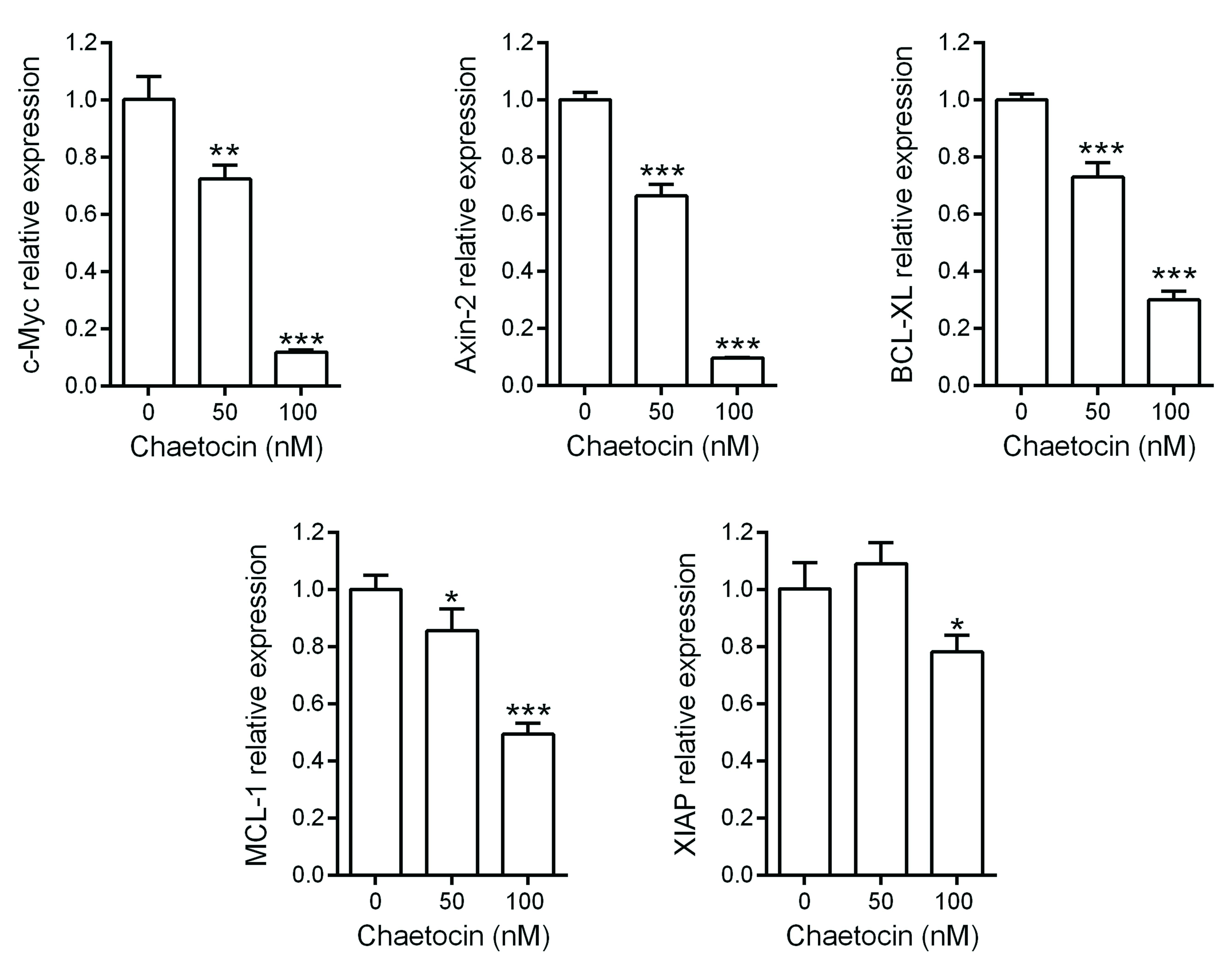

Supplement: Supplementary file 6 — Supplementary Fig.4 [file 41419_2019_2035_MOESM6_ESM.jpg]

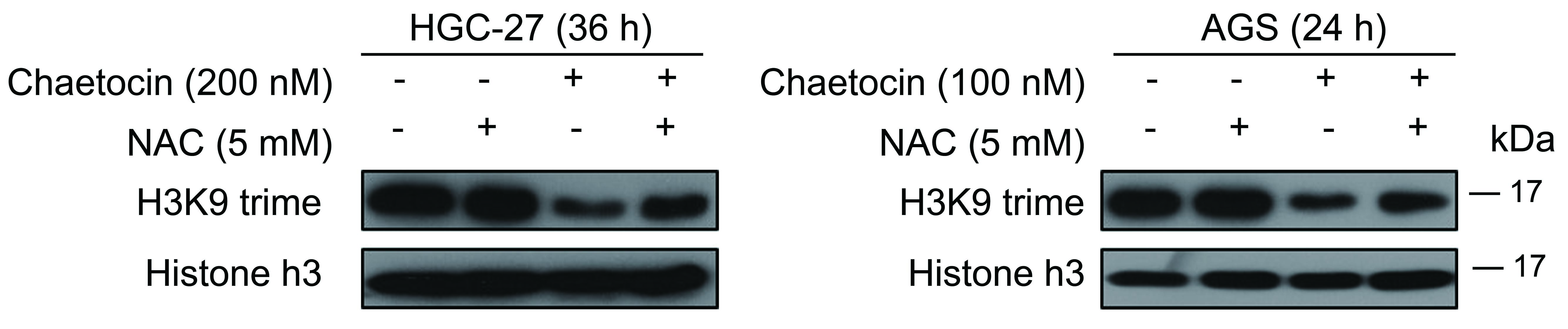

Supplement: Supplementary file 7 — Supplementary Fig.5 [file 41419_2019_2035_MOESM7_ESM.jpg]

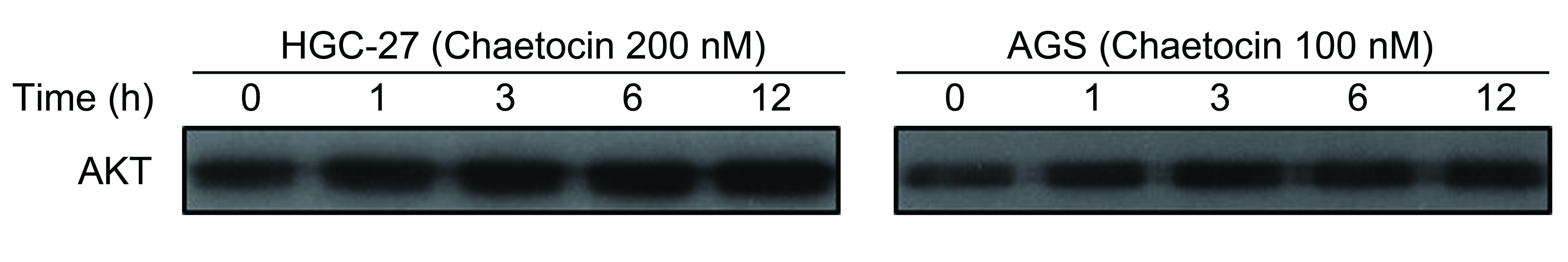

Supplement: Supplementary file 8 — Supplementary Fig.6 [file 41419_2019_2035_MOESM8_ESM.jpg]
